# Development and validation of an institutional nomogram for aiding aneurysm rupture risk stratification

**DOI:** 10.1038/s41598-021-93286-6

**Published:** 2021-07-05

**Authors:** QingLin Liu, Peng Jiang, YuHua Jiang, HuiJian Ge, ShaoLin Li, HengWei Jin, Peng Liu, YouXiang Li

**Affiliations:** 1 Department of Neurosurgery, Beijing Tiantan Hospital of Capital Medical University, Beijing, 100050 China; 2 Department of Interventional Neuroradiology, Beijing Neurosurgical Institute and Beijing Tiantan Hospital of Capital Medical University, Beijing, 100050 China; 3 Beijing Neurointerventional Engineering Center, Beijing, 100050 China

**Keywords:** Stroke, Stroke

## Abstract

Rupture risk stratification is critical for incidentally detected intracranial aneurysms. Here we developed and validated an institutional nomogram to solve this issue. We reviewed the imaging and clinical databases for aneurysms from January 2015 to September 2018. Aneurysms were reconstructed and morphological features were extracted by the *Pyradiomics* in python. Multiple logistic regression was performed to develop the nomogram. The consistency of the nomogram predicted rupture risks and PHASES scores was assessed. The performance of the nomogram was evaluated by the discrimination, calibration, and decision curve analysis (DCA). 719 aneurysms were enrolled in this study. For each aneurysm, twelve morphological and nine clinical features were obtained. After logistic regression, seven features were enrolled in the nomogram, which were SurfaceVolumeRatio, Flatness, Age, Hyperlipemia, Smoker, Multiple aneurysms, and Location of the aneurysm. The nomogram had a positive and close correlation with PHASES score in predicting aneurysm rupture risks. AUCs of the nomogram in discriminating aneurysm rupture status was 0.837 in a separate testing set. The calibration curves fitted well and DCA demonstrated positive net benefits of the nomogram in guiding clinical decisions. In conclusion, *Pyradiomics* derived morphological features based institutional nomogram was useful for aneurysm rupture risk stratification.

## Introduction

Intracranial aneurysms are pathological dilations at the primary bifurcations of intracranial vasculatures, with a prevalence of about 3.2% (95%CI 1.9–5.2%) of the adult population (mean age of 50 years) worldwide^1^. A recent study reported that the annual rupture risk was only 0.95% (95%CI 0.79–1.15%)^2^, demonstrating a relatively low rupture risk. However, once the aneurysm ruptures, the overall case fatality rate is 25 to 50%^3,4^, and the dependency rate is approximately 50% in survivors^5^. Fearing of the disastrous consequence after aneurysm rupture, many patients go for preventive treatment. However, the overall 30-day morbidity and mortality rate after treatment in patients without previous hemorrhage is 13.7% and 9.7%, in open surgical and endovascular groups, respectively^6^. These results often place the physicians in a dilemma of whether to treat an accidentally detected aneurysm. The solution is to screen out the most dangerous aneurysms for treatment and leave the relatively safe ones for conservation.

Great efforts have been paid for aneurysm rupture risk stratification. Size has been proposed as the most important predictive index. According to the International Study of Unruptured Intracranial Aneurysms (ISUIA), the 5-year cumulative rupture risks for aneurysms less than 7 mm in the anterior and posterior circulation without a previous hemorrhage was 0% and 2.5%, respectively^6^. This result indicates that small aneurysms (less than 7 mm) may possess a neglectable risk considering those from preventive treatment. However, in a retrospective study, small aneurysms (less than 7 mm) account for 38% of all ruptured aneurysms^7^, indicating the irrationality of only using the size in deciding whether an unruptured aneurysm should be treated or not.

Although controversies remain in different studies, patient-related clinical factors such as hypertension, smoking have been proven as risk factors for aneurysm rupture^1^. Based on the characteristics of the patients and the aneurysms, a well-documented PHASES scoring system has been developed, which enrolled the following determinants: Population, Hypertension, Age, Sex, Earlier history of subarachnoid hemorrhage (SAH) from another aneurysm and Site of the aneurysm^8^. The simplicity of this scoring system makes it the most popular model in daily clinical practice. However, this system exhibits an unsatisfactory clinical performance. In a study enrolling 100 consecutive ruptured aneurysms, 70% of the patients with aneurysmal SAH would exhibit a low rupture risk according to the PHASES score^9^. These results imply that the PHASES scoring system may have missed other important factors for aneurysm rupture risk stratification.

Other reports confirmed that irregularity was associated with aneurysm stability^7,10–12^. In the three-year risk assessment system from a Japanese cohort, irregularity was enrolled as an independent risk factor for aneurysm rupture^13^. However, in this system, aneurysm irregularity was only qualitatively dichotomized as with or without a daughter sac. Quantitative indexes such as size ratio (SR), flow angle (FA), height/width ratio (H/W ratio), aspect ratio (AR), undulation index (UI), ellipticity index (EI), and nonsphericity index (NSI) have also been proposed for aneurysms rupture risk stratification^10–12^. However, the low repeatability between raters or the complexity of calculation hindered their daily clinical usage. High reproducible quantitative morphological features are pressing needed.

Proposed by Lambin et al. in 2012, the term radiomics refers to the high-throughput extraction of quantitative imaging features from radiographic images^14^. These features involve descriptors of intensity distribution, spatial relationships between the various intensity levels, texture heterogeneity patterns, descriptors of shape, and of the relations of the tumor with the surrounding tissues^14^. Radiomics has provided valuable complementary information for decision support in clinical oncology. However, lacking standardization of both feature definitions and image processing makes the reproduction and comparison of results difficult^15^. To address this issue, an open Python program named *PyRadiomics* was released, with which radiomics features could be easily and quickly extracted, including morphological features^15^. These morphological features can delineate the irregularity of an object in a quantitative manner. The application of these morphological features for aneurysm rupture risk stratification has not been reported.

In this study, based on the clinical features of the patients and the quantitative morphological features of the aneurysm automatically extracted from *PyRadiomics,* we developed a nomogram to predict the rupture riks for an unruptured aneurysm*.* The consistency of the nomogram and PHASES score predicted aneurysm rupture risks were tested, and the performance of the nomogram was validated by discrimination, calibration, and DCA analysis.

## Methods

### Patient cohort and aneurysm acquisition

The data supporting the findings of this study are available from the corresponding author upon reasonable request. All patients were from the Beijing TianTan Hospital of Capital Medical University. This study was conducted by the Declaration of Helsinki and approved by the Ethics Committee of Tiantan Hospital affiliated to Capital Medical University (2018-0117/2018-09-06), and written informed consent was obtained from each patient before the operation. The cohort of patients enrolled was from January 2015 to September 2018. The inclusion criteria were: patients who had 3D digital subtraction angiography by Siemens Artis Zee System (Siemens Healthcare, Erlangen, Germany); a confirmed ruptured or unruptured diagnosis of the aneurysm; sufficient image quality for 3D vessel construction with no artifacts to accurately represent aneurysm and parent vasculature; saccular aneurysm; and available clinical charts. Aneurysms accompanied with other vascular abnormalities such as moyamoya disease, arteriovenous malformation, and arteriovenous fistula were excluded. A total of 719 aneurysms in 579 patients met the criteria were enrolled for analysis.

### Acquisition of clinical and morphological features

Methods in acquiring the clinical and morphological features have been reported in our previous study^16^. Clinical features enrolled in this study were those that have been reported as potential risk predictors for aneurysm rupture. They were gender, hypertension, hyperlipemia, diabetes, smoking and drinking status, and multiplicity and location of aneurysms^13,17–20^. These features were collected by reviewing the in-hospital medical records.

Concerning morphologic features, sectional 3D imaging data of the vessels in DICOM (Digital Imaging and Communications in Medicine) format from the Siemens Artis Zee workstation were imported to the Software 3D Slicer (version 4.8.0; http://www.slicer.org), and a threshold-based algorithm was used to reconstruct the 3D imaging of the vasculature. Then, the aneurysm was manually segmented from the parent vessel by two individual interventionalists (Liu QL, Ge HJ). For each aneurysm, the parent vessel and the segmented aneurysm were saved in NRRD (Nearly Raw Raster Data) format files of the same size. Subsequently, the two NRRD format files of an aneurysm were read by the program of *PyRadiomics* implemented in Python, and the measurements of twelve morphological features were automatically extracted for each aneurysm and exported into a file of EXCEL format^15^. These morphological features include the following: Compactness 1 (a measure of how compact the shape is relative to a sphere), Compactness 2 (a measure of how compact the shape is relative to a sphere), SurfaceArea (the total area of the shape), SurfaceVolumeRatio (the ratio of surface area to volume of a shape), Sphericity (a measure of the roundness of the shape relative to a sphere), SphericalDisproportion (the ratio of the surface area to the surface area of a sphere with the same volume), Maximum3DDiameter (the largest pairwise Euclidean distance between surface mesh vertices), Maxium2DDiameterSlice (the largest pairwise Euclidean distance between surface mesh vertices in the axial plane), Maximum2DDiameterColumn (the largest pairwise Euclidean distance between surface mesh vertices in coronal plane), Maximun2DDiameterrow (the largest pairwise Euclidean distance between surface mesh vertices in the sagittal plane), Elongation (a measure shows the relationship between the two largest principal components in the shape) and Flatness (a measure shows the relationship between the largest and smallest principal components in the shape). Detailed information on these features is available in the documentation for *PyRadiomics* (http://PyRadiomics.readthedocs.io/en/latest/).

### Development of the nomogram for aneurysm rupture risk stratification

All the aneurysms were randomly sampled into two separate groups, named the training and testing sets. The training set contained 70% of all aneurysms, and the testing set contained the rest 30% aneurysms. Data equilibrium between the training and testing set was tested. The training set was to select the features and determine their weights to construct the prediction nomogram, and the separate testing set was used to validate the performance of the nomogram. Features were compared between ruptured and unruptured aneurysms in the training set for selecting potential risk factors for aneurysm rupture. Features with *P* < 0.1 were enrolled in multiple logistic regression and backward stepwise regression was employed to determine the final model. Nomogram was plotted based on the results from multiple logistic regression with the rms and foreign packages in the software of R (R: A Language and Environment for Statistical Computing, R Core Team, R Foundation for Statistical Computing, Vienna, Austria, 2019, https://www.R-project.org).

### Feasibility evaluation of the nomogram

The PHASES score is the most widely accepted and applicated tool for assessing the rupture risks for an unruptured aneurysm^8,21^. To assess the feasibility of the nomogram for aneurysm rupture risk stratification, we tested the consistency of our nomogram predicted rupture risks with PHASES score in unruptured aneurysms. First, the nomogram predicted rupture risks, and the PHASES score of each unruptured aneurysm were calculated. Second, the correlation was tested between nomogram predicted rupture risks and the PHASES scores. Third, aneurysms were classified into low (PHASES score < 5), medium (PHASES score 5–9), and high rupture risk (PHASES score > 9) subgroups according to PHASES score. The nomogram predicted rupture risks were compared between these groups.

### Validation of the nomogram

Ten-fold cross-validation was performed to test the robustness of the model in the training set. The proportions of aneurysms with a high nomogram predicted rupture risk and high PHASES score in ruptured aneurysms were calculated. The discrimination capacity of the nomogram and PHASES system for aneurysms rupture status was exhibited as receiver operating characteristics curves (ROCs). AUCs were used to demonstrate their discrimination capacity. Calibration curves were plotted to compare the nomogram predicted with the actual rupture status. The net benefit of the nomogram and PHASES system was evaluated by decision curve analysis (DCA).

### Statistical analysis

Statistical analysis and figure plotting were conducted with the R software (R: A Language and Environment for Statistical Computing, R Core Team, R Foundation for Statistical Computing, Vienna, Austria, 2019, https://www.R-project.org). The student *t-*test or Wilcoxon test was used to compare the continuous morphological features between the training and testing set and between ruptured and unruptured subgroups in the training set according to the results from normality and variance equality tests. The Chi-square test was employed to compare the categorical clinical features between groups. Multiple logistic regression and backward stepwise regression were used to construct and refine the prediction model. Spearman correlation coefficient was calculated between the nomogram predicted rupture risks and PHASES score, and the significance of the correlation was tested with cor.test() function in R. Comparison of the nomogram predicted rupture risks between the low, medium and high PHASES score groups were conducted with Kruskal–Wallis rank-sum test. Subsequent multiple comparisons between every two groups were conducted with Dunn's test if the difference was significant between these three groups (*P* < 0.05). Calibration analysis for the nomogram was tested by the Hosmer–Lemeshow test. The main packages used in this study mainly include pROC, ggplot2, caret, DescTools, foreign, rms, boot, and nricens.

## Results

### Patient cohort and aneurysm features

A total of 719 aneurysms in 579 patients (385 females and 194 males) were enrolled in this study, of which 503 were unruptured, and 216 were ruptured. The median maximum diameter of the aneurysms was 6.137 mm, ranging from 2.268 to 18.927 mm. As to the location of the aneurysms, 52.6% (378/719) located at the anterior or posterior communicating artery and posterior circulation, 14.0% (101/719) located at the middle cerebral artery, and the rest 33.4% (240/719) located at the internal carotid artery. This cohort of aneurysms was randomly sampled into the training (504 aneurysms, 147 ruptured) and testing (215 aneurysms, 69 ruptured) set. The comparison of the features between the training and testing set were summarized in Table 1. As the table showed, all of the features were well balanced between these two sets (*P* > 0.05 for each feature).

**Table 1:** Comparison of the features between the training and testing set.

	Training set (N = 505) Median(IQR)	Testing set (N = 214) Median(IQR)	*P*
**Morphological features**			
Compactness1	0.037 (0.035, 0.039)	0.037 (0.035, 0.039)	0.830
Compactness2	0.492 (0.427, 0.545)	0.493 (0.438, 0.543)	0.830
SurfaceArea	67.469 (41.920, 112.527)	75.048 (41.098, 123.606)	0.578
SurfaceVolumeRatio	1.864 (1.474, 2.358)	1.780 (1.398, 2.424)	0.535
Sphericity	0.789 (0.753, 0.817)	0.790 (0.759, 0.816)	0.830
SphericalDisproportion	1.267 (1.224, 1.328)	1.265 (1.226, 1.317)	0.830
Maximum3DDiameter	6.134 (4.586, 7.750)	6.153 (4.616, 8.091)	0.861
Maximum2DDiameterSlice	5.115 (4.082, 6.843)	5.139 (4.102, 7.181)	0.524
Maximum2DDiameterColumn	5.182 (4.001, 6.803)	5.354 (4.001, 6.832)	0.844
Maximum2DDiameterRow	5.385 (4.167, 6.990)	5.440 (4.236, 7.337)	0.807
Elongation	0.776 (0.664, 0.861)	0.768 (0.677, 0.857)	0.603
Flatness	0.633 (0.538, 0.732)	0.638 (0.554, 0.714)	0.992
**Clinical features**			
Age(> 60)	197 (39.0%)	79 (36.9%)	0.657
Sex(Female)	329 (34.9%)	151 (29.4%)	0.186
Hypertension(yes)	283 (56.0%)	109 (50.9%)	0.24
Hyperlipemia(yes)	63 (12.5%)	32 (15.0%)	0.437
Diabetes(yes)	66 (13.1%)	26 (12.1%)	0.829
Smoking(yes)	122 (24.2%)	37 (17.3%)	0.054
Drinking(yes)	115 (22.8%)	38 (17.8%)	0.161
Multiple(yes)	183 (36.2%)	79 (36.9%)	0.930
**Aneurysm location**			0.170
Acom/Pcom/Post	272 (53.9%)	106 (49.5%)	
MCA	75 (14.9%)	26 (12.1%)	
ICA	158 (31.3%)	82 (38.3%)	
Ruptured aneurysm	157 (31.2%)	59 (27.6%)	0.394

*IQR* interquartile range, *Acom* anterior communicating artery, *Pcom* posterior communicating artery, *Post* posterior circulation, *MCA* middle cerebral artery, *ICA* internal carotid artery.

### Construction of the nomogram

**Figure 1:**
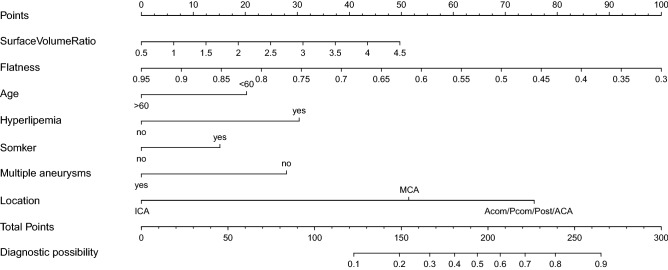
Nomogram for predicting aneurysm rupture risk. To calculate the rupture risk of an aneurysm, first determine the value for each feature by drawing a vertical line from that feature to the points scale. Then sum up all the individual values and draw a vertical line from the total points scale to the probability at the Diagnostic Probability line to obtain the rupture risk estimates.

In the training set, morphological and clinical features were compared between the ruptured (147 aneurysms) and unruptured groups (357 aneurysms). The results were summarized in Table 2. Of the 21 features, 15 were selected as candidates for multiple logistic regression (*P* < 0.1). They were Compactness1, Compactness2, SurfaceVolumeRatio, Sphericity, SphericalDisproportion, Elongation, Flatness, Age, Sex, Hypertension, Hyperlipemia, Smoking, Drinking, Multiple aneurysms, and Location of the aneurysm. These Features were taken into multiple logistic regression and backward stepwise regression was employed to solve colinearity and construct the final model. The model was plotted as a nomogram shown in Fig. 1. Features enrolled in the nomogram included: SurfaceVolumeRatio, Flatness, Age, Hyperlipemia, Smoking, Multiple aneurysms, and Location of the aneurysm.

**Table 2:** Comparison of the features between ruptured and unruptured aneurysms in the training set.

	Ruptured (N = 147) Median(IQR)	Unruptured (n = 357) Median(IQR)	*P*
**Morphological features**			
Compactness1	0.036 (0.033, 0.038)	0.038 (0.036, 0.040)	< 0.001*
Compactness2	0.453 (0.391, 0.526)	0.506 (0.452, 0.554)	< 0.001*
SurfaceArea	66.053 (38.421, 112.653)	67.541 (43.068, 111.855)	0.612
SurfaceVolumeRatio	1.939 (1.531, 2.563)	1.833 (1.430, 2.308)	0.050^†^
Sphericity	0.768 (0.731, 0.807)	0.797 (0.767, 0.822)	< 0.001*
SphericalDisproportion	1.302 (1.239, 1.367)	1.255 (1.217, 1.303)	< 0.001*
Maximum3DDiameter	6.273 (4.565, 8.234)	5.894 (4.537, 7.556)	0.222
Maximum2DDiameterSlice	5.115 (3.873, 7.088)	5.115 (4.102, 6.695)	0.904
Maximum2DDiameterColumn	5.113 (4.001, 6.976)	5.17 (3.959, 6.642)	0.559
Maximum2DDiameterRow	5.290 (3.931, 7.318)	5.318 (4.167, 6.931)	0.996
Elongation	0.693 (0.587, 0.800)	0.808 (0.712, 0.875)	< 0.001*
Flatness	0.570 (0.453, 0.659)	0.661 (0.581, 0.743)	< 0.001*
**Clinical features**			
Age(> 60)	45 (30.6%)	150 (42.0%)	0.022^‡^
Sex(Female)	60 (40.8%)	111 (31.1%)	0.046^‡^
Hypertension(yes)	89 (60.5%)	184 (51.5%)	0.081^†^
Hyperlipemia(yes)	37 (25.2%)	25 (7.0%)	< 0.001*
Diabetes(yes)	19 (12.9%)	39 (10.9%)	0.627
Smoking(yes)	54 (34.4%)	68 (19.5%)	< 0.001*
Drinking(yes)	45 (30.6%)	60 (16.8%)	< 0.001*
Multiple(yes)	36 (24.5%)	160 (44.8%)	< 0.001*
**Aneurysm location**			< 0.001*
Acom/Pcom/Post	117 (79.6%)	142 (39.8%)	
MCA	21 (14.3%)	52 (14.6%)	
ICA	9 (6.1%)	163 (45.7%)	

*IQR* interquartile range, *Acom* anterior communicating artery, *Pcom* posterior communicating artery, *Post* posterior circulation, *MCA* middle cerebral artery, *ICA* internal carotid artery.

*P < 0.001, ^†^*P* < 0.1, ^‡^*P* < 0.05.

### Feasibility evaluation of the nomogram in predicting aneurysm rupture risks

**Figure 2:**
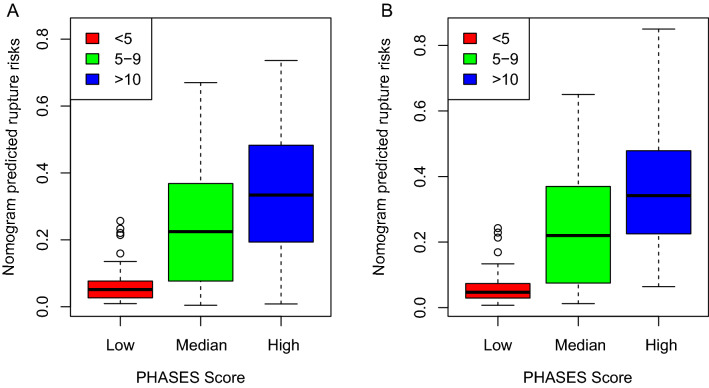
The nomogram predicted risks were compared across categories of PHASES score. Unruptured aneurysms were classified into low (PHASES < 5), medium (PHASES 5–9), and high (PHASES > 9) rupture risk subgroups. The medium nomogram predicted rupture risks were compared between these groups in the training (**A**) and testing (**B**) set, respectively.

Rupture risks were predicted with the nomogram for all aneurysms, and the correlation of the predicted risks and the PHASES scores were calculated and tested in unruptured aneurysms. Both in the training and testing set, the nomogram predicted risks exhibited high consistency with the PHASES score. The estimated correlation coefficient was 0.532 (95% CI 0.453–0.602, *P* < 0.001) and 0.543 (95% CI 0.419–0.649, *P* < 0.001) for the training and testing set, respectively. Unruptured aneurysms were classified into low (PHASE score < 5), medium (PHASES score 5–9), and high (PHASES score > 9) rupture risk subgroups. The nomogram predicted rupture risks were compared between these subgroups in both the training (Fig. 2A) and testing (Fig. 2B) sets. In the training set, the median nomogram predicted rupture risks elevated with the increased PHASES score, with significant differences between all the three groups (*P* < 0.001), and between the low and medium (*P* < 0.001), the low and high (*P* < 0.001), and the medium and high (*P* = 0.004) PHASES score groups. Similar results were seen in the testing set, with significant differences between all the three groups (*P* < 0.001), and between the low and medium (*P* < 0.001), the low and high (*P* < 0.001), and the medium and high (*P* = 0.019) PHASES score groups.

### Validation of the nomogram

**Figure 3:**
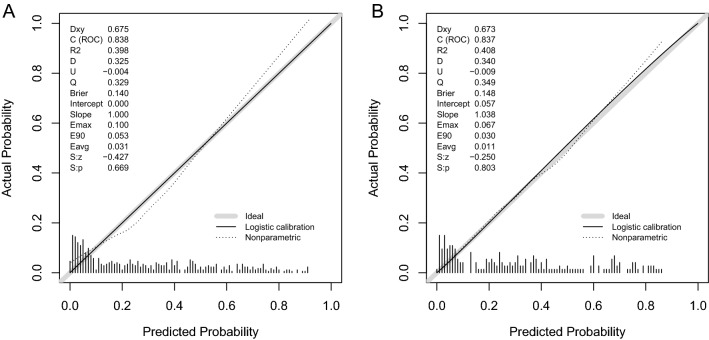
Calibration of the nomogram. The nomogram predicted rupture risks demonstrated high consistency with the actual rupture status in the training (**A**, *P* = 0.669) and testing (**B**, *P* = 0.803) sets.

**Figure 4:**
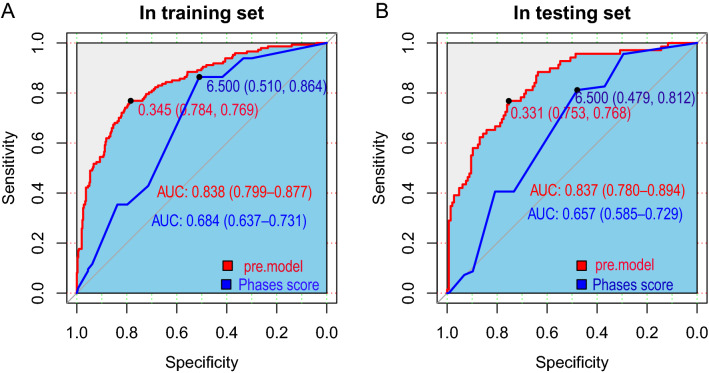
Discrimination of ruptured and unruptured aneurysms by the nomogram and PHASES score. ROCs were built to compare the performance of the nomogram and PHASES score in discriminating ruptured and unruptured aneurysms by comparing the AUCs in the training (**A**) and testing (**B**) sets. AUC: areas under the curve; Pre.model: the nomogram predicted risks; Phases score: the PHASES score; ROC: Receiver operating characteristic curve.

**Figure 5:**
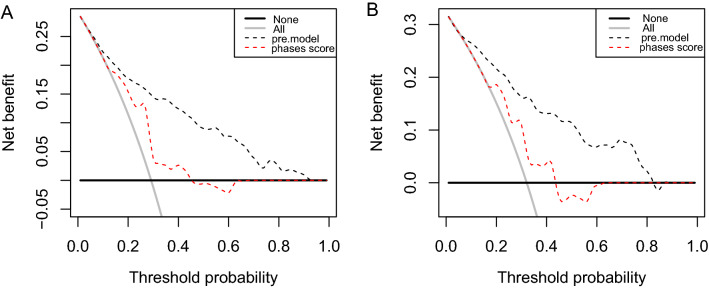
Decision cure analysis of the nomogram. Net benefit was compared between the nomogram and PHASES score instructed treatment. Both in the training (**A**) and testing (**B**) sets, the nomogram instructed treatment gained an even higher net benefit than the PHASES system in our series. All: all aneurysms were treated; None: no aneurysm was treated; Phases score: treatment decision was instructed by PHASES score; Pre.model: treatment decision was instructed by the nomogram.

The calibration plot (Fig. 3) showed favorable agreement between our monogram predicted rupture risks and actual rupture status in the training (Fig. 3A, *P* = 0.669) and testing set (Fig. 3B, *P* = 0.803). For all ruptured aneurysms, the proportion of aneurysms with a PHASES score higher than 7 is 57.1% (84/147) and 59.4% (41/69) in the training and testing set, respectively. The range of the nomogram predicted rupture risks is from 0.014 to 0.919 in the training set, and 0.029 to 0.866 in the testing set. The optimal cutoff value for discriminating ruptured and unruptured aneurysms for our nomogram is 0.345 in the training set (Fig. 4A). The proportion of ruptured aneurysms with a nomogram predicted rupture risks higher than 0.345 is 76.9% (113/147) and 72.5% (50/69) in the training and testing set, respectively. The adjusted ten-fold cross-validation estimate of prediction error was 0.144 in the training set, demonstrating the high robustness of the model. In the training set, the AUC of the nomogram in discriminating aneurysm rupture status was 0.838 (95% CI 0.799–0.877), which was higher than that of the PHASES system (0.684, 95% CI 0.637–0.731, *P* < 0.001) (Fig. 4A). In the testing set, the AUC of the nomogram in discriminating aneurysm rupture status was 0.837 (95% CI 0.780–0.894), which was also higher than that of the PHASES system (0.657, 95% CI 0.585–0.729, *P* < 0.001) (Fig. 4B). DCA revealed that both the nomogram and PHASES score provided a net benefit to the none or all strategy in both the training (Fig. 5A) and testing (Fig. 5B) set.

## Discussion

It has reached a consensus that ruptured aneurysms should be treated as soon as possible to avoid disastrous rebleeding, but the treatment of unruptured aneurysms remains controversial. Although great progress has been made on both endovascular and open surgical techniques, the morbidity and mortality rates from preventive treatment remain unneglectable. This raises the issues of screening the real dangerous aneurysms for preventive treatment. In this study, we developed a nomogram for aneurysm rupture risk stratification, which exhibited high consistency with the PHASES system and provided a net benefit than the none or all treatment strategy in our series.

Released in 2014, the PHASES system enrolled the largest prospective cohort to predict the 5-year aneurysm rupture risks^8^. The easy application and high-level evidence make it one of the most popular scoring systems in daily clinical practice, but its performance sometimes was not so satisfying^22^, implying some important risk factors may be missed. Candidate baseline risk factors enrolled in this system included date of inclusion, age, sex, history of SAH, smoking status, hypertension, number of aneurysms, the maximum diameter of aneurysms, and aneurysm location^8^. As a widely accepted rupture risk factor^7,10–12^, morphology of the aneurysm was not considered in this system. In another famous scoring system for assessing the 3-year aneurysm rupture risks in a Japanese cohort, the morphology of the aneurysm was enrolled qualitatively^13^. The determination of whether an aneurysm was irregular and its severity was always subjective. Quantitative indexes such as SR, FA, H/W ratio, AR, UI, EI, and NSI have been widely studied for aneurysm rupture risk stratification^10–12^, with the shortcomings of low repeatability between raters or the complexity of calculation. In this study, we introduced morphological indexes extracted from a radiomics program named *Pyradiomics*, which was implemented in Python. With this program, morphological indexes including the size and irregularity descriptors could be easily and automatically extracted, and high consistency could be reached between raters^15^. These descriptors could delineate the size and irregularity of the aneurysm quantitatively. These portraits made these indexes more reliable and gave the potential of depicting the regularity of an aneurysm quantitatively.

The PHASES score system was developed from a large prospective study^8^, which supplied high-level evidence for clinical practice. Data used to develop the nomogram in our study came from a respective database, which provides a relatively inferior evidence level to a prospective study. A prospective patient cohort with long time clinical and imaging follow-up is ideal to develop the model for predicting the rupture risks of an unruptured aneurysm. However, the establishment of such a natural cohort is to some extent unreasonable. For example, for patients with relatively high rupture risks according to the existing stratification systems such as the PHASES and the Japanese 3-year scoring system, a conservation strategy would place them in a dangerous situation and encounter an ethical debate. On the other hand, only keeping the so-called low-risk aneurysms for observation would generate a severe selection bias and would make the comparison of the actual natural history and the preventive treatment-related risks less reliable. Another concern for our study is that we used the post-rupture morphology to predict the rupture status, and the post-rupture morphology has been reported to be different from their pre-rupture state in small series^23^. However, the actual growth process is irregular and discontinuous, which results in periods with and without aneurysm growth and with high and low risks of rupture^24^. Thus, morphology at a specific time may also insufficient for predicting aneurysm stability. Furthermore, individual characteristics of the patients may have significant effects on the growth speed of unruptured aneurysms^25^. Based on the clustering theory that individuals sharing similar features may harbor similar properties, we took the post-rupture morphology for constructing the prediction model. To evaluate the feasibility of our nomogram, we firstly tested the correlation of our nomogram predicted rupture risks and PHASES scores for the unruptured aneurysms in both the training and testing sets. The correlation coefficient was 0.532 and 0.543 with a significant correlation in the training (*P* < 0.001) and testing (*P* < 0.001) set, respectively, demonstrating a close positive correlation between the nomogram and PHASES predicted rupture risks. Furthermore, the nomogram predicted rupture risks elevated with the low, medium, and high PHASES score subgroups (Fig. 2). These results confirmed the feasibility of our nomogram in predicting aneurysm rupture risks, despite it came from a retrospective cohort and enrolled post-rupture morphology.

Risk factors in our nomogram include SurfaceVolumeRatio, Flatness, Age, Hyperlipemia, Smoking, Multiple aneurysms, and Location of the aneurysm, which were slightly different from the PHASES score system. SurfaceVolumeRatio and Flatness were irregularity indexes that were ignored by the PHASES system at the initial design. SurfaceVolumeRatio and Flatness could reflect the irregularity of the object quantitatively. Our result was consistent with the previous study that the more irregular the higher rupture risks^13^. Size was not enrolled in our nomogram but was deemed as the most important risk factor in the PHASES system. The absence of size in our nomogram might come from the patient selection bias, as this cohort came from in-hospital patients most of whom received preventive treatment. The selection of the patients was greatly influenced by the Chinese expert consensus for endovascular treatment of intracranial aneurysms (2013), which recommends that asymptomatic aneurysms larger than 5 mm should be considered for preventive treatment^26^. Older age was a ‘protective factor’ in our nomogram but ranked as a risk factor in the PHASES system^8^. This controversy may also come from the unique constitution of the patients. As the biggest neuro-intervention suit in China, most patients with ruptured aneurysms came from the nearby regions of the city. However, most patients with unruptured aneurysms may come from all over our country, and the higher imaging examination frequency for other vascular lesions in older patients inevitably resulted in a higher detection rate in these small populations, resulting in a higher average age for the patients with unruptured aneurysms. Similarly, multiplicity is thought to be a risk factor for aneurysm rupture and multiple aneurysms are prone to be preventively treated^26^. However, multiplicity is statistically ‘protective’ for aneurysm rupture in our results. This reflects the selection preference of multiple aneurysms for preventive treatment in our cohort (44.8%). These disparity of aneurysm multiplicity and patient age only reflect the unique patient constitution in our institution, but not the real-world risk factor. We did not conclude that aneurysm multiplicity and older age were protective factors for aneurysm rupture. On contrary, these results reflect our concerns for higher rupture risks in older patients and multiple aneurysms. Again, we emphasize that the prediction nomogram was institutional. Other risk factors enrolled in our nomogram were consistent with the previous studies^8,13,17–20^.

Several ruptured aneurysm series have been used to retrospectively assess the predicting value of the PHASES score system^9,27^. In our cohort, 57.1% (84/147) ruptured aneurysms in the training set and 59.4% (41/69) ruptured aneurysms in the testing set featured a PHASES score higher than 7, which is higher than that of the previous report^27^. When discriminated by the optimal cutoff value of 0.345 from the training set, 76.9% (113/147) and 72.5% (50/69) of ruptured aneurysms were ranked as high rupture risk in the training and testing set respectively, demonstrating a high consistency with the actual status. Calibration curves also revealed high consistency between the nomogram estimates and the actual rupture status (Fig. 3, *P* = 0.669 and 0.803 in the training and testing set, respectively). Discrimination capacity for ruptured and unruptured aneurysms was demonstrated by constructing the ROCs. As shown by Fig. 4, AUCs of our nomogram was 0.838 and 0.837 in the training and testing set, respectively, which was even higher than the PHASES system (0.684 and 0.657, *P* < 0.001, respectively). DCA was initially used by Vickers and Elkin as a new analytical technique, incorporating the clinical consequences of a decision, to quantify the clinical usefulness of a prediction model^28^. As shown in Fig. 5, the net benefit from decision instructed by our nomogram or PHASES system was higher than all or no treatment strategy in both the training and testing sets, and an even greater net benefit was gained from the nomogram than the PHASES system. However, this doesn't mean that our nomogram is superior to the PHASES score system, as the testing set is an internal validating set for our nomogram and an external validating set for the PHASES system. The unique patient and aneurysm constitution may also hinder the generalization of the PHASES score in our institution. Furthermore, the retrospective design of our study renders an inferior evidence level to the PHASES score. This study was not to develop a prediction model that parallel to or even to replace the PHASES score, but to provide more information for aneurysm rupture risk stratification besides the PHASES score.

### Limitations

This study features certain limitations. First, the nomogram was developed on a cohort from a single institution with a single Race. The generalizability of the nomogram may be limited as the distinct patient constitution of our single center. We emphasize the term of an institutional nomogram to encourage the development of an institutional-specific model for better predicting performance. Second, the data for developing the nomogram was retrospective, and the time-dependent rupture risks could not be calculated from the nomogram. Third, we also ignored some features that contribute to aneurysm ruptures, such as the family history of aneurysm^29^, previous SAH from another aneurysm^8^, hemodynamic parameters^30^, and aneurysm wall enhancement patterns^31^. Fourth, although the traditional generalized linear regression model was adopted in this study, generalized estimating equations or mixed-effect models may be more appropriate.

## Conclusions

Despite the limitations, conclusions can still be obtained from this study. First, *Pyradiomics* derived morphological features could be used for aneurysm rupture risk stratification. Second, institutional specific nomogram could be developed and adopted as a useful tool for aiding rupture risk stratification for an incidentally detected intracranial aneurysm.

## Data Availability

Data supporting this study were available from the corresponding author on reasonable request.
